# Knowledge and perception about the welfare and mistreatment of dogs in Brazil

**DOI:** 10.1371/journal.pone.0302317

**Published:** 2024-04-19

**Authors:** Gabriela Ferreira Siano, Camila Stefanie Fonseca de Oliveira, Felipe Gaia de Sousa, Suzane Lilian Beier, Adriane Pimenta da Costa-Val

**Affiliations:** 1 Department of Veterinary Clinics and Surgery, Veterinary School, Federal University of Minas Gerais, Belo Horizonte, Minas Gerais, Brazil; 2 Department of Veterinary Preventive Medicine, Veterinary School, Federal University of Minas Gerais, Belo Horizonte, Minas Gerais, Brazil; Oswaldo Cruz Foundation Rene Rachou Institute: Fundacao Oswaldo Cruz Instituto Rene Rachou, BRAZIL

## Abstract

Animal welfare encompasses the overall well-being of an animal, spanning both its physical and mental health, assessable through potential measurements. It stands in contrast to mistreatment, which involves actions, direct or indirect, that endanger an animal’s well-being. This study sought to appraise the factors influencing the Brazilian population’s understanding of dog welfare and mistreatment. The survey questions were adapted from the Animal Welfare Examination Protocol, utilized by veterinarians to evaluate suspected passive abuse cases in dogs. Out of 1377 responses, 1353 were valid and analyzed. Among the 19 assessed indicators, 15 demonstrated an adequate response rate surpassing 90% of all 1353 responses. However, for three questions related to comfort, a smaller yet notable percentage of responses were only minimally adequate. Moreover, in one question within the comfort assessment, 186 participants (13.74%) provided inadequate responses. This implies that these people could potentially subject animals to a state of low Animal Welfare. Lack of knowledge emerged as a potential root of passive abuse, specifically negligence. In the assessment of nutritional indicators, water supply and quality received unanimous adequate responses. In evaluating comfort perceptions, significant associations were noted between gender, dog ownership, family income, and responses regarding resting surface. Regarding health indicators, the majority responded appropriately. Female gender and dog ownership correlated with providing the appropriate response, while not owning a dog was associated with minimally adequate responses. In the context of comfort indicators, "Hitting the Dog" also demonstrated an association with gender, with females tending towards appropriate responses. Given the lack of significant correlation between educational levels and the most suitable responses, it underscores the urgency of implementing environmental education programs in schools with a focus on animal protection.

## Introduction

In Brazil, 44.3% of households have at least one dog, as reported by the Brazilian Institute of Geography and Statistics—IBGE (2015) [1]. Additionally, according to Brazilian Association of the Pet Products Industry (2023), the number of dogs in Brazil in the year of 2022 was 67.8 millions, with growing up 3.5% in relation of 2021 [2]. It is the responsibility of animal guardians to provide necessary care for their pets [3]. However, some guardians may mistreat animals through neglectful, violent, or cruel actions, compromising their well-being [4–6]. The association between animal and/or object hoarding, recognized as a psychological disorder, should be highlighted. This condition leads to the indiscriminate collection of animals from the streets and their confinement in inadequate living conditions, potentially resulting in low levels of animal welfare [7]. There are variations in the profile of the Brazilian population concerning knowledge about well-being, responsible ownership, and abuse. This can be attributed, for instance, to the country’s vast geographical expanse and diverse cultures. However, studies dedicated to assessing the level of knowledge, implementation, as well as the factors influencing, regulating, and monitoring these issues are generally limited, which hampers the acquisition of a comprehensive understanding of the population’s profile. Consequently, that remains an underdiagnosis in evaluating the population’s dynamics concerning responsible pet ownership, well-being, and abuse. Thus, studies adopting this perspective are imperative. The dissemination and application of information on this pertinent subject, particularly within the context of the Brazilian population, still require broader outreach [8, 9].

The term "animal welfare" (AW) refers to an animal’s quality of life at a specific point in time, encompassing both its physical and psychological state [10, 11]. The attitudes or omissions of owners have the potential to either enhance or compromise the welfare of the animals under their care [12]. Quality of welfare comprises concepts referring to the quality of life offered to animals, so that they have sufficient conditions for well-being, including “good food”, “good housing”, “good health” and “appropriate behavior” [13]. One approach to assessing animal welfare is through the consideration of the Five Freedoms: Freedom from hunger and thirst, Freedom from discomfort, Freedom from pain, injury, or disease, Freedom to express normal behavior, and Freedom from fear and distress [14]. The initial conception of the Five Freedoms for farm animals dates back to 1992 and was established by the Farm Animal Welfare Council. Its primary objective was to ensure the well-being of animals and mitigate the risks of adverse situations [15]. Over the years, there have been refinements, and in light of the increasingly close interactions between animals and humans, a new adaptation was deemed necessary in 2020.

In the most recent adaptation [16], additional information concerning physiology and affective experiences was incorporated, with a specific focus on well-being and strategies to enhance it. A comprehensive evaluation of animal welfare encompasses five domains, with four concentrating on physical and functional aspects: nutrition (Domain 1), physical environment (Domain 2), health (Domain 3), and behavioral interactions (Domain 4) [16]. The ultimate domain (Domain 5 –Mental state/Affects) addresses the psychological state of animals, envelop the emotions linked to deprivation [16, 17]. Domain 1 –nutrition pertains to the quality of food and water provided to animals, Domain 2 –physical environment involved the physical conditions and atmosphere in which animals reside, and Domain 3 –health addresses the implications of injuries and diseases on the overall health status of animals [16]. Domain 4 –behavioral interactions focuses on behavioral adaptability in response to unforeseen circumstances, further divided into three subcategories based on the nature of the interactions. Lastly, Domain 5 –mental state surround the psychological well-being of animals in relation to stress, pain, and anxiety [16]. Animal welfare is inversely related to animal abuse, which includes neglect or omission and intentional cruelty [18].

Animal cruelty comprehend deliberately inflict unnecessary pain, suffering, distress, and even death upon animals [5]. Humans have control over critical elements like water, food, living conditions, and socialization for domestic animals. Neglect is characterized by a failure to provide essential necessities for a healthy life, such as food, water, veterinary care, cleanliness, and proper sanitation, resulting in deteriorated physical conditions. Neglect is the most common form of animal abuse investigated by authorities and may arise from ignorance, poverty, or extenuating circumstances, often being passive and unintentional [4, 19]. The study by Penaforte et al. (2022) [20] near Belo Horizonte, Brazil, highlights that dogs roaming semi-freely or as strays is linked to issues of irresponsible ownership. They emphasize that many current problems, including disease transmission and accidents, are linked to loose animals on the streets, which could be mitigated with responsible ownership. The authors advocate for holding legal guardians accountable and promoting awareness of responsible ownership, especially in urban areas. They also point out that less urbanized regions may face additional challenges due to less regulated and supervised animal management practices.

Due to the myriad possible scenarios, accurately assessing animal welfare is a complex process that requires precise tools for various situations [21]. The Animal Welfare Examination Protocol (AWEP) is one such tool designed for evaluating animal welfare in suspected abuse cases in Brazil. It comprises four indicator categories inspired by the concept of the Five Freedoms. Each category can be classified as inadequate (severe resource limitations), minimally adequate (resources available with some limitations), or adequate (resources appropriately available). The overall animal welfare status is determined by the combination of these four associated categories, which may be rated as low or very low. If physical aggression is involved, it is described as animal abuse [14, 22].

Understanding the primary care needs of animals is crucial for the population. While most dog owners are knowledgeable about their pets’ dietary requirements, other aspects like behavior and health still require attention. Investigating the Brazilian population’s perception of dog care is vital in devising practical measures to prevent animal abuse [23]. This study aims to address the variation in knowledge levels regarding animal welfare and abuse among different socio-demographic groups in Brazil. The scarcity of research on this topic underscores the importance of evaluating the knowledge and perception of basic dog needs within these groups.

## Materials and methods

The study was approved by the National Health Council (Conselho Nacional de Saude): Plataforma Brasil, CAAE 89680318.6.0000.5149

Participants agreed to participate and signed the Consent Form as they began to answer the questionnaires, applied from August.1^st^ 2021 to October, 30^rd^, 2021. The study involved the administration of an electronic questionnaire to individuals aged 18 and above, which contained objective questions derived from the Animal Welfare Examination Protocol (AWEP) used by veterinarians in Brazilian official service to assess suspected cases of passive abuse in dogs [24]. The AW indicators were categorized into four groups: nutritional (Table 1), comfort (Table 2), health (Table 3), and behavior (Table 4). Due to difficulties encountered in understanding the environmental and psychological states of animals by participants during a pilot study, these domains were evaluated through questions framed under the category of "comfort".

**Table 1:** Questioning, classification, percentage of answers in nutritional related indicators.

Nutritional Indicator	Option in questionnaire	Classification of the answer	Percentage of answers	Associations with sociodemographic variables (p<0.05)
*Frequency of water* *supply*	Water must always be readily available.	Proper	98.82%	The unanimity of the response did not allow testing association
	Water should be given one or more times a day, with no problem with lack of it at certain times.	Minimally adequate	0.96%	
	Water should be offered to the dog when he seems to be thirsty.	Inadequate	0,035%	
	Putting water for the dog is unnecessary	Inadequate	0.035%	
	I do not rknow how to answe	I do not know how to answer	0.015%	
*Quality of supplied water*	It must be fresh, clean, and drinkable	Proper	99.19%	The unanimity of the response did not allow testing association.
	It does not need to be clean and drinkable. It can be, for example, from the rain.	Inadequate	0.22%	
	I do not know how to answer	I do not know how to answer	0.59%	
*Food quality*	Food	Proper	97.71%*	Gender (p = 0.146) Education (p = 0.000) Income (p = 0067) Dog guarding (p = 0.652)
	Homemade diet	Proper	39.62*	Gender (p = 0.000) Schooling (p = 0.988) Income (p = 0.972) Guard of khans (p = 0128)
	Food scraps	Inadequate	5.84%	Gender (p = 0.000) Schooling (p = 0.158) Family Income (p = 0.593) Dog guarding (p = 0.083)
	I do not know how to answer	I do not know how to answer	0.44%	Not applicable
*Frequency of food offer*	Food must be freely available throughout the day	Proper	95.86%	
	Food should be offered every day, at least twice a day	Proper		Gender (p = 0.000) Schooling ((p = 0.01) Income (p = 0.788) Dog guarding (p = 0.001)
	Food should be offered every day, once a day	Minimally adequate	3.33%	
	It is not necessary to put food for the dog, because he looks for it himself when he feels hungry	Inadequate	0.15%	
	I do not know how to answer	I do not know how to answer	0.67%	
*Cleaning of Canisters*	It is essential for the dogs’ health to clean the bowls frequently	Proper	94.60%	Gender (p = 0.000) Income (p = 0.465) Education (p = 0.000) Dog guarding (p = 0.692)
	It is essential, for the health of the dogs, that the bowels are cleaned when they already have dirt, leftover, and lousy smell	Minimally adequate	4.90%	
	It is enough to change the water and food without having to clean the bowls	Inadequate	0.22%	
	I do not know how to answer	I do not know how to answer	0.22%	

Note

* Responders may choose more than one option.

**Table 2:** Questioning, classification, percentage of answers comfort related indicators.

Comfort Indicator	Option in questionnaire	Classification of the answer	Percentage of answers	Associations with sociodemographic variables (p<0.05)
*Shelter*	It is necessary to have a house or shelter for the dog to protect itself	Proper	98.37%	The unanimity of the response did not allow testing association
	It is not necessary to have a house or shelter for the dog to protect itself, as it knows how to hide	Inadequate	0.89%	
	I do not know how to answer	I do not know how to answer	0.74%	
*Surface for rest* *	Solid surface such as mattress or rug	Proper	94.09%	Gender (p = 0.040) Education (p = 0.000) Yield (p = 0.469) Dog guarding (p = 0.000)
	Box paper Grass	Minimally adequate	36.37%	Gender (p = 0.958) Education (p = 0.273) Income (p = 0.095) Dog guarding (p = 0.009)
	Soil Cement	Inadequate	12.05%	Gender (p = 0.131) Schooling (p = 0.342) Income (p = 0.026) Dog guarding (p = 0.692)
	I do not know how to answer	I do not know how to answer.	2.59%	Gender (p = 0.198) Schooling (p = 0.142) Income (p = 0.926) Dog guarding (p = 0.000)
*Stuck on rope/chain*	The dog cannot be tied to a rope or chain.	Proper	77.24%	Gender (p = 0.000) Schooling (p = 0.200) Income (p = 0.223) Dog guarding (p = 0.000)
	The dog can be kept on a rope or chain for a period, if he is let out at night or walked around to move around	Minimally adequate	19.96%	
	The dog can be tied to a rope or chain day and night	Inadequate	0.67%	
	I do not know how to answer	I do not know how to answer	2.14%	
*Stuck in stall/kennel*	The dog cannot remain trapped in these places	Proper	56.17%	Gender (p = 0.000) Education (p = 0.016) Income (p = 0.699) Dog guarding (p = 0.0722)
	The dog can be trapped day and night, if he goes for walks to move around. The dog can be trapped during the day, but released at night	Minimally adequate	35.33%	
	The dog can be trapped all day and night, without having to go out	Inadequate	0.07%	
	I do not know how to answer	I do not know how to answer	6.43%	
*Available space*	The place must be wide, allowing free circulation	Proper	61.64%	Gender ((p = 0.008) Schooling (p = 0.001) Income (p = 0.612) Dog guarding (p = 0.367)
	The location may be limited, but it should at least allow the dog to move and take short runs	Minimally adequate	37.18%	
	The size of the place where the dog is not important, it can be small and limited	Inadequate	0.44%	
	I do not know how to answer	I do not know how to answer	0.74%	
*Bowls cleaning*	It is essential for the dogs’ health to clean the bowls frequently	Proper	98.82%	The unanimity of the response did not allow testing association
	It is essential, for the health of the dogs, that the bowels are cleaned when they already have dirt, leftover, and lousy smell	Minimally adequate	1.03%	
	I do not know how to answer	I do not know how to answer	0.15%	

Note

* Responders may choose more than one option.

**Table 3:** Questioning, classification, percentage of answers in health related indicators.

Health Indicator	Option in questionnaire	Classification of the answer	Percentage of answers	Association with sociodemographic variables (p<0.05)
*Fleas and ticks*	It causes discomfort and diseases, it must be treated preventively, so as not to appear	Proper	91.50%	Gender (p<0.05) Education (p<0.05) Dog guarding (p<0.05)
	It causes discomfort and disease; it must be treated when it appears	Minimally adequate	8.06%	
	It’s normal, every dog has it, and nothing needs to be done	Inadequate	0.07%	
	I do not know how to answer	I do not know how to answer	0.37%	
*Worms or other parasites in the intestine*?	It causes discomfort and diseases, it must be treated preventively, so as not to appear	Proper	92.17%	Gender (p<0.05) Education (p<0.05) Dog guarding (p<0.05)
	It causes discomfort and disease; it must be treated when it appears	Minimally adequate	7.24%	
	It’s normal, every dog has it, and nothing needs to be done	Inadequate	0.07%	
	I do not know how to answer	I do not know how to answer	0.52%	
*Application of vaccines*	In addition to the free vaccines offered by the city, the dog must receive vaccines against other diseases	Proper	91.20%	Gender (p<0.05) Schooling (p<0.05) Dog guarding (p<0.05)
	The free vaccines offered by the city hall are sufficient	Minimally adequate	5.76%	
	No vaccinations are needed for dogs	Inadequate	0.07%	
	I do not know how to answer	I do not know how to answer	2.96%	
*How walks are done*	They are important for the dog and must always be done on a leash to avoid escapes and accidents	Proper	91.48%	Education (p<0.05)
	They are important for the dog and can be done without a guide, freely, supervised by the owner	Minimally adequate	6.66%	
	They are important for the dog, which can freely roam the street without supervision and then return to its home	Inadequate	0.45%	
	I do not know how to answer	I do not know how to answer	1.05%	

**Table 4:** Questioning, classification, percentage of answers in behavior related indicators.

Behavior Indicator	Option in questionnaire	Classification of the answer	Percentage of answers	Association with sociodemographic variables (p<0.05)
*Social interaction*	The dog needs to have contact with other animals and people	Proper	98.82%	The unanimity of the response did not allow testing association
	It’s okay for a dog to be in isolation without having contact with other animals and people.	Inadequate	0.59%	
	I do not know how to answer	I do not know how to answer	0.59%	
*Dog’s activities*	The dog needs toys and games with the tutor to exercise and not stress.	Proper	98.08%	The unanimity of the response did not allow testing association
	Toys and games are unnecessary	Inadequate	0.96%	
	I do not know how to answer	I do not know how to answer	0.96%	
*Need for walks*	They are important for the dog and can be done without a guide, freely, supervised by the owner They are important for the dog and must always be done with a leash to avoid escapes and accidents	Proper	97.71%	Gender (p = 0.019) Schooling (p = 0.001) Family income (p = 0.024) Dog guard (p = 0.072)
	They are important for the dog, which can freely go out into the street without supervision and then return to its home They are unnecessary for the dog	Inadequate	1.26%	
	I do not know how to answer	I do not know how to answer	1.03%	
*Hit the dog*	Hitting a dog is always wrong, it shouldn’t be done.	Proper	83.07%	Gender (p = 0.000) Education (p = 0.000) Income (p = 0.980) Dog guarding (p = 0.168)
	Sometimes it is necessary to hit a dog to teach and correct it, for example not to climb on the sofa or urinate in the wrong place It’s okay to hit a dog, because he’s an animal	Inadequate	13.74%	
	I do not know how to answer	I do not know how to answer	3.18%	

Each category included subcategories (found in the first column of each table). These indicators were transformed into questions (second column of each table), and the responses were classified as adequate, minimally adequate, or inadequate (third column of each table) based on AW principles [14]. An option for "I do not know how to answer" was also provided. In total, nineteen indicators were assessed.

Snowball sampling, also known as chain sampling, was employed. Is a method for selecting participants in a research study. In this type of sampling, initial participants are recruited in a non-random manner and are then asked to refer other individuals who may fit the study’s selection criteria [25, 26]. Weekly comparisons of response rates were conducted to determine the saturation point, which occurred when responses stabilized, with variations within 0–0.1% [27, 28]. Data from respondents under 18 years old or those who did not disclose their age were excluded from the analysis.

The Chi-square statistical test was used to examine associations between response patterns and sociodemographic factors such as gender, education, family income, and dog ownership. This was except for food quality and resting surface, where the test was applied individually for each response. In cases where responses reached a consensus of 98% or higher, indicating near unanimity, further analysis was deemed unnecessary.

When associations were identified between variables, standardized residuals were analyzed to pinpoint the factor directly influencing the choices (residual greater than 1.96). A significance level of 5% was maintained for all statistical analyses [29].

## Results and discussion

From 1377 responses received, 1353 valid responses were analyzed because data from 24 respondent under 18 years old or those who did not provide age information were excluded.

### Socio-demographic factors

The average age of participants was 38.3 ± 13.2 years. The 18–39 age group was the most prevalent (812/1353, 60.0%), followed by participants aged 40 to 59 years (427/1353, 31.6%), and those aged 60 years or older (113/1353, 8.4%). The majority of respondents identified as women (1020/1353, 75.4%), followed by men (325/1353, 24.0%), while a small percentage identified as another gender or did not provide information (8/1353, 0.6%). These demographic distributions in terms of age and gender differ [30] from the overall Brazilian population, indicating that the web-based questionnaire may not have captured a representative sample specific to the Brazilian context, in contrast to experiences in other countries such as U.S. [31].

Most participants (908/1353, 67.1%) reported having completed higher education, while 418 (30.9%) stated they had completed high school. Only twenty participants (1.5%) mentioned completing basic education, and five (0.5%) did not provide information about their educational background. In a study conducted by Penaforte et al. (2022) [20] in Divinópolis/Minas Gerais, Brazil, to evaluate the population’s perception of animal welfare, approximately 38.5% of respondents had incomplete elementary school. There is a noticeable discrepancy in educational attainment between the sample and the broader Brazilian population [32]. In the general Brazilian population, only 11.27% have completed higher education, 24.56% have finished high school, and 14.65% have completed elementary school. A similar trend was observed in income distribution, with 184 participants (13.60%) reporting earnings up to two minimum wages, which is significantly lower than the distribution within the Brazilian population, where 80.9% earn up to two minimum wages. However, within the income bracket of two to ten minimum wages, the sample’s proportion (792/1353, 58.5%) exceeds that observed in the Brazilian population, which is 16.8%. This trend is also reflected in the bracket above ten salaries (260/1353, 19.2%), compared to 2.3% in the broader Brazilian population [32]. These differences can be attributed to the sampling method, which relied on participants sharing the research with their contacts, resulting in a sample that closely resembled one another.

The majority of participants (1028/1353, 77.53%) reported owning dogs, which aligns with findings from other studies [33]. This could be attributed to a heightened interest in participating in the survey among dog owners, likely due to the subject matter.

### Answers

The existing literature has highlighted a lack of knowledge [12, 24] as a potential contributor to passive abuse, particularly negligence, although no direct studies have examined this association. In a study conducted by Monsalve et al. (2018) [34] in the city of Pinhais, Brazil, out of the 118 cases evaluated, animal abuse was found in 76.3% of the cases, all of which involved negligence. Approximately 40.7% of the animals were classified as having low welfare, 35.6% as very low, 5.9% as regular, 12.7% as high, and 5.1% as very high. The data described by Monsalve et al. (2018) [34] emphasize the importance of assessing the husbandry conditions of animals within homes and providing legal guardians with guidance on well-being, quality of life, and the provision of appropriate breeding conditions.

This study facilitated an assessment of the knowledge and awareness of individuals within this sample regarding fundamental dog care, which is integral to their well-being. The overall level of knowledge was deemed satisfactory, despite the prevalent occurrence of neglectful maltreatment in cases typically investigated in Brazil [19]. It’s worth noting that the sampling method, based on the Snowball technique and reliant on voluntary participation, may have influenced this perception. As a result, the observed participation may have been skewed towards individuals already interested in the subject matter, potentially possessing greater prior knowledge [31]. Consequently, studies examining knowledge in different populations could yield varying levels of understanding on the subject. Another potential explanation for this discrepancy could be that in this research, a small percentage responded with only minimal adequacy or inadequacy to certain items. This subgroup of the population may represent individuals lacking essential knowledge, inadvertently contributing to maltreatment through negligence. This is significant as studies on incidence are typically based on abuse complaints, i.e., cases with a higher likelihood of confirmation. Additionally, it’s plausible that other factors, such as domestic violence and abuse of children and the elderly, could contribute to negligence-type maltreatment alongside the lack of knowledge. Among the 19 indicators evaluated, 15 showed a high level of appropriate responses, exceeding 90% of all 1353 responses. However, for 3 questions related to comfort, a smaller but notable percentage (between 20 and 37%) of responses were only minimally adequate. Additionally, in one question within the comfort assessment, 186 participants (13.74%) provided inadequate responses. This suggests that these individuals might inadvertently compromise animal well-being, as their responses indicate a significant limitation in certain resources.

Regarding nutritional indicators (Table 1) the items’ water supply and the water quality obtained near unanimity of the adequate answers. This knowledge contrasts with the incidence of maltreatment due to negligence as lack of food and water, even leading to malnutrition and cachexia [18, 34]. This difference can be explained by the fact that this study was carried out in the general population and not in complaints of mistreatment, which already means a greater possibility of finding a situation of low well-being. In addition, there is a need to consider that other factors can lead to deprivation of nutritional conditions, such as social vulnerability. For food quality, the ration response did not present an association with any of the sociodemographic factors evaluated, which indicates that income and education do not seem to be a limiting factor for considering the supply of nutritionally balanced food. The use of a natural diet made at home is associated with the female gender. Despite the small proportion found, the item leftover food showed an association (p<0,05) with the male gender, and was indicated as appropriate by 5.84% of responders, which differs from what was found in a study that evaluated undergraduate students’ perception of animal welfare, in which 42% claimed to offer the same food they eat to their animals [33]. This fact could be justified by the type of approach since what the participant considers appropriate in the present research was questioned. In contrast, in the previous work, the conduct carried out in practice was addressed. In addition, in the study mentioned earlier, undergraduate students may not represent the principal caregivers of these animals and may have responded based only on what they eventually do. Regarding the frequency of food supply, there was an association (p<0.05) between gender and dog custody; being female was associated with the adequate response. This may be related to the fact that women are the dogs’ primary caregivers and are primarily responsible for feeding the house, which can influence the choice of healthier food. Despite the small number of these responses, having a dog was positively associated with the adequate response, but not having a dog was associated with the minimally adequate responses. Despite the shallow frequency, the inappropriate option regarding the frequency of food supply was related to gender (p<0.05) and was associated with male gender (p<0.05). Concerning cleaning the bowls, the minimally adequate response corresponded to a small percentage of responses (4.9%), and there was no association with sociodemographic factors. As for the other sociodemographic factors, there was no association as well.

In the category of comfort indicators (Table 2), 50% of the questions received unsuitable answers scoring above 90%. Regarding the surface area for resting items, there were associations with gender, dog ownership, and family income. Being female and owning a dog were directly related to providing appropriate responses, while not owning a dog was associated with minimally adequate responses or uncertainty in answering. On the other hand, family income between two and ten minimum wages was related to inadequate responses. In the study by Monsalve et al. (2018) [34], the authors observed that the most significant deficiencies were found in behavior and comfort indicators, which were inadequate in 50% and 63.6% of the cases, respectively. These findings support the present study and underscore the need to raise public awareness about the quality of life provided to animals, particularly in terms of confinement in ropes, chains, stalls, and kennels, as well as the layout of the living space.

As for the question about keeping the dog attached to a rope/chain, there was an association (p<0.05) with gender and guarding dogs, with female gender and having dogs having an influence on the appropriate response, and male gender and not having a dog on the minimally adequate response and did not know how to respond. As for being confined in a pen/kennel, there was an association with gender (p<0.05), being that the female was related with the adequate answer and the male gender showed an association with the minimally adequate answer, whereas education showed an association, but only the uninformed category was associated with the option I do not know to answer. For the space available, an association was found (p<0.05) with gender, but only the category not informed/others and unable to answer showed a relationship. The population’s adequate knowledge of comfort needs contrasts with what is found in cases of complaints of abuse, in which it is observed that compromised comfort factors are frequent findings in investigations. Many complaints involve a lack of available shelter or resting areas, or when there are, they are insufficient, and the cleaning conditions of the environment are precarious [35]. Income did not influence other items besides resting surface; schooling did not influence any item or the number of dogs. The fact that the category that presented the worst level of knowledge is that of comfort coincides with what has been observed in surveys of complaints of mistreatment of animals [19, 34]. This demonstrates that, although knowledge about comfort indicators has been satisfactory, there are still individuals who do not consider it essential for the environment to present adequate conditions for the comfort of dogs. Cultural issues can determine the normalization of leaving dogs in conditions of limited space, and restriction of movement, without protection from the weather and surface to rest, demonstrating the need for awareness actions to change this perception.

Regarding the health indicators (Table 3), all four evaluated indicators showed more than 90% of adequate answers. In terms of flea and tick prevention, there was a significant association (p<0.05) with gender, education level, and dog ownership. Having a dog was associated with providing an adequate response, while not having a dog was linked to a minimally adequate response. In the study by Penaforte et al. (2022) [20], the authors observed a high risk of dogs not remaining in their homes, especially male dogs with high levels of tick infestation, free access to wooded areas. The presence of ticks and inadequate control strategies may contribute to the spread of diseases, not only among animals but also to humans in close contact. Regarding the prevention of worm infestation, an association (p<0.05) was found with gender, education level, and dog ownership. Higher education level was associated with providing an appropriate response, while high school education was associated with a minimally adequate answer. Having a dog was linked to an adequate response, while not having a dog was associated with a minimally adequate response. In terms of vaccine administration, there was a significant association (p<0.05) with gender, education level, and dog ownership. Female gender and owning a dog were related to providing an adequate response, while not having a dog was associated with a minimally adequate response.

Despite the satisfactory level of knowledge about vaccines found, the attitudes of dog owners differ from the findings of a study conducted by da Silva et al. (2020) [33], where 70% of the participants vaccinated their pets only during city hall campaigns. Additionally, according to IBGE (2015) [1], in households with a dog or cat, only 75.4% (24.9 million) had all their animals vaccinated against rabies in the last 12 months. Furthermore, as Hammerschmidt et al. (2017) [18] suggests, evidence demonstrates a low rate of deworming and vaccination in abused dogs.

Regarding the way walks are conducted, the majority (91.84%) responded adequately about how walks are done, and there was an association with the level of formal education. Although there was a small proportion (0.45%) of inadequate responses regarding how walks should be done, these responses were associated (p<0.05) with income, family and education level. Having an income of up to two minimum wages and an average education level were associated with this response. The way walks are carried out is intertwined with health indicators since allowing dogs to walk freely in the street without supervision (inadequate) or releasing them with supervision (minimally adequate) can put them at risk of accidents and fights, leading to injuries, pain, and even death, as identified as a cause of death in a study on reports of animal abuse [35].

Regarding the responses to the Behavior indicators shown in Table 4, of all evaluated items, three obtained more than 90% of adequate responses. The need to go out was associated with education and income (p<0.05), with complete high school education being positively related to adequate response and family income above ten minimum wages showing an association with inadequate response. The latter resembles the one found by Marinelli et al. (2007) [36], where dogs reside in large houses with a garden, which would be correlated with higher income in Brazil. They are rarely taken for walks, which prevents social contact with other dogs and people. Despite people’s adequate knowledge of the need for dogs to have contact with other animals and people unanimously, social isolation has been a relevant finding in cases of reports of mistreatment, which may be explained by cultural issues related to the difference between the assessed sample and other populations.

In the item hitting the dog, there was an association with gender p<0.05, and being female was related to the adequate response. It was observed that 13.7% of respondents responded inappropriately, which was considered appropriate for beating dogs to teach or correct. This answer was associated (p<0.05) with gender, and being male was an essential factor for this answer to be chosen (residue >+1.96). This indicator was associated with schooling p<0.05. However, when analyzing its residue, there was only a positive relationship between the uninformed level of schooling and the inadequate answer. Thus, there was no influence of income and schooling on this item. For the classification parameters of mistreatment according to BEA indicators, when a category of indicators is inadequate, it frames the animal’s situation as low well-being, configuring mistreatment. In addition, physical aggression toward the animal alone is enough to classify the case as mistreatment. This relationship between considering it acceptable to hit an animal and the male gender could be reflected in the fact that in cases of active maltreatment when there is an intention to harm an animal, there are greater chances of the agent involved being a man, which was found in several works in which complaints of mistreatment of animals were studied [23].

From the perspective of sociodemographic factors, in this research, it was observed that the female gender was an essential factor for adequate response in several items within the indicators, which may be related to the fact that women are generally the principal caregivers of companion animals and present a greater degree of attachment or connection to these animals, which was observed both by Ramón et al. (2010) [31] and Bures et al. (2019) [37]. A study conducted by Henry et al. (2009) [38] suggested that women are more empathetic towards animals than men, and even have a broader perception of what could be considered cruelty to animals. This could imply more excellent knowledge on the subject and greater interest in participating in this research, which may explain the predominance of females in the sample and the level of knowledge manifested by the studied population.

Conversely, males contributed significantly (p<0.05 and residue >+1.96) to minimally adequate and inadequate responses to several items, particularly regarding hitting dogs. The male gender is a significant factor in mistreatment, cruelty, abuse, and neglect. In this way, there is a connection between animal and human [39]. Gomes et al. (2021) [40] describe that men are more likely to be cruel to animals, and this may be associated with educational factors. The phenomenon that explains why men are more associated with cases of animal abuse and neglect still requires further investigation, although it may be influenced by feelings of power, lack of family structure or care, and/or insensitivity. Several studies have observed that male animal abusers show less affection towards companion animals and are more likely to communicate with them solely through commands and threats [19, 35, 39, 40]. Furthermore, these abusers often consider pets as property and use punishment as a means of correction or teaching [41].

Studies have demonstrated a link between domestic violence and violence against animals. However, the male gender can also be associated with passive abuse. Studies based on reports of animal mistreatment complaints indicate that the majority of individuals reported are men, with most cases involving neglect. Additionally, it has been highlighted that in households with instances of domestic violence, the level of care provided to dogs tends to be lower [22]. Another study revealed that withholding essential care from a dog can be used as a form of threat or violence against women [41]. In the present research, it was found that the overall knowledge level of the general population is desirable. However, for several items, the male gender was identified as a factor contributing to inadequate and minimally adequate answers.

Regarding education level, there was no influence of the highest level of education on knowledge about dog care. As found by Marinelli et al. (2007) [36], being a graduate had a negative impact on general care knowledge, including veterinary care, with knowledge increasing up to the average education level but decreasing for dogs belonging to individuals with a higher education level. However, in the work of Ramón et al. (2010) [31], a higher education level was associated with a higher knowledge score about pet care. Although active maltreatment was associated with lower education levels, inadequate responses regarding hitting dogs were not associated with educational level [19].

The sociodemographic factor of family income did not consistently impact the population’s knowledge level. An income range of up to two minimum wages was associated with inadequate responses regarding how walks should be conducted. However, for other indicators, there was no association with family income. It’s worth noting that the snowball sampling model may have had a greater impact on the responses than the participants’ sociodemographic characteristics. Encouraging voluntary participation through the researchers’ and participants’ network of contacts may have led to the selection of similar individuals who had a greater interest in the subject and, therefore, a higher level of knowledge. This can be exemplified by the fact that owning a dog was correlated with several appropriate responses, while not owning a dog impacted minimally adequate responses. Dog ownership indicates a greater interest in the subject and, consequently, greater knowledge. Therefore, studies conducted in other populations and with different sampling methodologies can provide additional information about people’s knowledge of animal welfare and mistreatment. Furthermore, investigating causes beyond lack of knowledge, such as gender and situations of social vulnerability, can contribute to highlighting factors related to neglect-type maltreatment of dogs, especially in Brazil.

## Conclusions

Overall, the knowledge of the evaluated population regarding animal welfare factors, responsible ownership, and mistreatment of dogs was considered satisfactory. Females demonstrated a higher number of adequate responses compared to males. However, the male gender was associated with minimally adequate responses to comfort indicators and inadequate responses to behavior indicators. Family income and education level did not show a consistent association with the population’s knowledge level. Further studies are necessary to evaluate and analyze the profile of the population concerning well-being, responsible ownership, and mistreatment, particularly in the context of the Brazilian population where concrete research on the described situation is scarce. The findings of this study, despite its limitations, provide insights into the studied profile and suggest that the population, despite having satisfactory knowledge on the subject, should receive continuous education on responsible custody. It is also important to recognize that subsequent generations may approach the topic similarly to their parents due to influences, observations, and close parental contact.

## Supporting information

S1 Data (XLSX)
